# Impact of early-life weight status on urinary tract infections in children: a nationwide population-based study in Korea

**DOI:** 10.4178/epih.e2021005

**Published:** 2020-12-29

**Authors:** Hyung Eun Yim, Kyung Do Han, Bongseong Kim, Kee Hwan Yoo

**Affiliations:** 1 Department of Pediatrics, Korea University Ansan Hospital, Ansan, Korea; 2 Department of Pediatrics, Korea University College of Medicine, Seoul, Korea; 3 Department of Statistics and Actuarial Science, Soongsil University, Seoul, Korea; 4 Department of Pediatrics, Korea University Guro Hospital, Seoul, Korea

**Keywords:** Adiposity, Body weight, Malnutrition, Pediatrics, Urinary tract infections

## Abstract

**OBJECTIVES:**

We aimed to evaluate the association between early-life weight status and urinary tract infection (UTI) risk in children.

**METHODS:**

A nationwide study was conducted using Korean National Health Screening (NHS) data and National Health Insurance Service data. A sample cohort was selected using data from the 2014 and 2015 NHS for infants and children (4-71 months) and followed up until the end of 2017. Participants were divided into 4 groups (underweight, normal weight, overweight, and obese) based on the weight-for-age (< 2 years) or body mass index (≥ 2 years). Hazard ratios (HRs) with 95% confidence intervals (CIs) for developing UTIs, cystitis, and acute pyelonephritis (APN) were calculated using a Cox proportional hazard model.

**RESULTS:**

Of 1,653,106 enrolled children, 120,142 (7.3%) developed UTIs, cystitis, and APN during follow-up. The underweight, overweight, and obese groups had higher risks of UTIs than the reference group after adjusting for age, sex, birth weight, and preterm birth. Between 2 years and 6 years of age, boys with underweight had a high risk of UTI and APN, while girls with overweight and obesity revealed elevated risks of UTIs, cystitis, and APN. The HRs for APN in boys with underweight and in girls with obesity were 1.46 (95% CI, 1.03 to 2.07) and 1.41 (95% CI, 1.13 to 1.75), respectively, after adjusting for age, sex, birth weight, and preterm birth. The incidence of APN did not decrease with age in underweight and obese children aged 2-6 years.

**CONCLUSIONS:**

Children with underweight, overweight, and obesity may be at high risk for UTIs.

## INTRODUCTION

Achieving and maintaining a healthy weight during childhood is significant for the overall health and growth of children [1]. A high amount of body fat in children places them at risk of serious health problems, and being underweight leads to various health concerns as well. Considering that the adipose tissue is significant for immune responses, a lack or excess of adipose tissue may affect the immune system, leading to greater vulnerability to infections [2]. In an observational chart review study, underweight children were more frequently hospitalized from the emergency department with respiratory infections than the normal-weight children [1]. Undernourished preschool children showed higher risks of both infections and infection-related morbidity in an Indian National Family Health Survey database [3]. Further, obesity in school-aged children was associated with a 5.29-fold higher bronchitis rate and 1.79-fold higher use of antibiotics [4]. An inpatient United States database of subjects aged 2-20 years also showed that the risk of urinary tract infections (UTIs) was significantly higher in obese females [5]. Likewise, a U-shaped association between body mass index (BMI) and the infection rate in adults has been reported in several studies [2,6].

UTIs are among the most common causes of acute illness in childhood [7,8]. More than 1 million children with UTIs visit clinicians annually in the United States, and approximately 50,000 children with UTIs are admitted per year, with inpatient costs > US$180 million [9,10]. It is also estimated that approximately 7% of girls and 2% of boys experience a UTI in the first 6 years of life and that 2.1% of girls and 2.2% of boys have a UTI before 2 years of age [9,11]. The worldwide prevalence of childhood obesity has been increasing, and that of childhood underweight still remains high [12,13]. Although associations between the weight status of children and UTI prevalence have been documented in the severely underweight or obese individual category [5,14,15], to the best of our knowledge, no comparable analysis has yet explored the associations of UTI incidence with a comprehensive set of BMI categories, ranging from underweight to obese, among children. Population-based epidemiology studies regarding UTIs in children are also unavailable in Korea, although a few retrospective population-based studies have been published based on epidemiological data on UTIs in other countries [16,17]. Therefore, in this study, we aimed to identify the associations between being underweight, overweight, and obese and developing UTIs, cystitis, and acute pyelonephritis (APN) in 1,653,106 Korean children, using data from the National Health Screening (NHS) program for infants and children and the National Health Insurance Service (NHIS). This is the first nationwide population-based study on UTIs using anthropometric data from the NHS program for infants and children, which was launched in November 2007 across Korea.

## MATERIALS AND METHODS

### Data sources and study design

We used nationwide data from the National Health Information Database (NHID) of the NHIS and from the NHS for infants and children in Korea. The NHID provided by the NHIS contains records of healthcare utilization and prescriptions [18]. The NHS for infants and children in Korea is a type of population surveillance system, which includes history-taking, physical examinations, anthropometric measurements, and questionnaires with anticipatory guidance [19]. Through the NHS for infants and children, 7 sets of tests are conducted in the following age groups: 4 to 6 (first), 9 to 12 (second), 18 to 24 (third), 30 to 36 (fourth), 42 to 48 (fifth), 54 to 60 (sixth), and 66 to 71 (seventh) months. Our nationwide study was performed using data from the NHS for infants and children aged 4-71 months recorded between January 1, 2014 and December 31, 2015 and from the NHID recorded between January 1, 2014 and December 31, 2017. A sample cohort was first selected from the 2014 and 2015 NHS for infants and children who were then followed up through 2017. Based on the data from the NHS, the children were categorized into the following 4 groups: underweight, normal, overweight, and obese. For these 4 groups, the incidence of UTIs, cystitis, and APN was determined according to the International Classification of Diseases, 10th revision (ICD-10) system using the data from the NHID.

### Group criteria

The underweight, normal, overweight, and obese groups were defined based on the 2017 Korean National Growth Charts for children and adolescents, namely, weight-for-age charts for boys and girls aged 4-24 months and BMI-for-age charts for boys and girls aged 2-6 years [20]. The criteria for underweight, normal weight, overweight, and obesity were defined as weight-for-age or BMI-for-age in < 5th, in the 5th to < 85th, in the 85th to < 95th, and in the ≥ 95th percentiles for age and sex.

### Study outcomes

**Figure 1.**
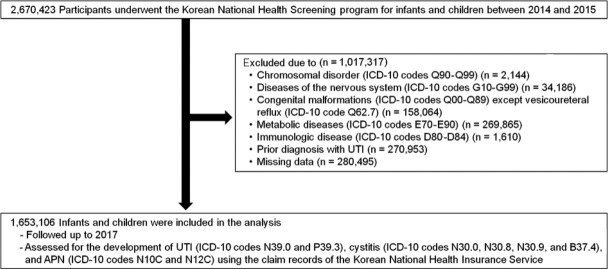
Flow chart of study enrollment. ICD-10, International Classification of Dis eases, 10th revision; UTI, urinary tract infection; APN, acute pyelonephritis.

We recorded UTIs, cystitis, and APN using the claim records of the NHIS until the end of 2017. Infants and children were included if they (1) had received an NHS examination for infants and children at least 1 time between 2014 and 2015 and (2) were NHIS subscribers supported by their guardians. The term “UTI” included diagnosis codes for cystitis (ICD-10 codes N30.0, N30.8, N30.9, and B37.4), APN (ICD-10 codes N10C and N12C), as well as UTIs (ICD-10 codes N39.0 and P39.3). We excluded children with records of (1) a previous history of UTI, cystitis, and APN before the NHS examination for infants and children, (2) systemic illnesses (chromosomal disorders [ICD-10 codes Q90-Q99], nervous system diseases [ICD-10 codes G10-G99], congenital malformations [ICD-10 codes Q00-Q89] except vesicoureteral reflux [ICD-10 code Q62.7], metabolic diseases [ICD-10 codes E70-E90], and immunologic diseases [ICD-10 codes D80-D84] according to the Korean ICD-10 system), and (3) missing values in baseline characteristics and covariates (Figure 1).

### Statistical analyses

The statistical analysis was performed using SAS version 9.4 (SAS Institute Inc., Cary, NC, USA). Categorical variables were compared between groups with the chi-square test, and continuous variables were compared using analysis of variance. Incidence rates (IRs) were calculated per 1,000 person-years. The association between weight-for-age or BMI-for-age and the development of UTIs, cystitis, and APN was estimated with hazard ratios (HRs) and 95% confidence intervals (CIs) using the normal weight group as the reference group. Cox proportional hazard model analysis was used to estimate the unadjusted (model 1) and adjusted HRs (models 2 and 3) for the associations. To control for confounding factors, we adjusted for age, sex, birth weight, and preterm birth. Data are presented as mean values with standard deviations for continuous variables and as proportions for categorical variables. All results were considered significant if the pvalue was < 0.05.

### Ethics statement

This study was approved by the Institutional Review Board of Korea University Ansan Hospital (No. 2019AS0025), and the requirement of obtaining informed consent was waived as this study involved a retrospective review of the medical records from anonymized participant data. This work was also carried out in accordance with the Declaration of Helsinki.

## RESULTS

### Patient characteristics

Among 2,670,423 children who underwent NHS examinations for infants and children between 2014 and 2015, 1,653,106 were enrolled and followed-up until the end of 2017 (Figure 1). The distribution of children with abnormal weight was as follows: 2.8%, underweight; 11.5%, overweight; and 6.4%, obesity. During follow-up, a total of 120,142 (7.3%) patients were diagnosed with UTIs (n=94,185, 5.7%), cystitis (n=16,716, 1.0%), and APN (n=9,241, 0.6%) among the 1,653,106 infants and children. The mean age, sex distribution, history of preterm birth, birth weight, and the incidence of UTIs, APN, and cystitis were significantly different among groups (p< 0.001). Weight status was associated with the total number of cases of UTIs, APN, and cystitis without adjustment for confounding variables (Table 1).

### Hazard ratios for urinary tract infection, cystitis, and acute pyelonephritis according to body weight status

Among the 1,653,106 infants and children who were analyzed, being underweight, overweight, and obese was associated with an elevated risk of UTIs. The overweight and obese children showed higher HRs for cystitis and APN. The HR for APN in obese children was 1.34 (95% CI, 1.24 to 1.44) without adjustment and 1.16 (95% CI, 1.08 to 1.25) with adjustment for age, sex, birth weight, and preterm birth. Between 4 months and 2 years of age, infants who were underweight showed an elevated risk of UTIs without adjustment (HR, 1.09; 95% CI, 1.03 to 1.16) or with adjustment (HR, 1.10; 95% CI, 1.04 to 1.17) for age, sex, birth weight, and preterm birth. Between 2 years and 6 years of age, children who were underweight, overweight, and obese showed an elevated HR for UTIs with adjustment for the confounding factors. In particular, the obese group showed higher HRs for cystitis and APN, while the overweight group had a higher HR for cystitis. After adjusting for age, sex, birth weight, and preterm birth, the HR for APN in obese children aged 2-6 years was 1.30 (95% CI, 1.08 to 1.57) relative to the normal-weight children (Table 2).

### Hazard ratios for urinary tract infection, cystitis, and acute pyelonephritis according to body weight status and sex

In both age groups (< 2 and ≥ 2 years old), boys with underweight showed trends for elevated HRs for UTIs, cystitis, and APN. In particular, underweight boys aged 2-6 years had a higher risk of UTIs (HR, 1.10; 95% CI, 1.01 to 1.19) and APN (HR, 1.46; 95% CI, 1.03 to 2.07) than their normal-weight counterparts after adjusting for age, sex, birth weight, and preterm birth. In contrast, in girls aged 2-6 years, the overweight and obese groups showed elevated HRs for UTIs, cystitis, and APN, respectively. The HRs for UTI, cystitis, and APN in obese girls were 1.22 (95% CI, 1.15 to 1.31), 1.37 (95% CI, 1.21 to 1.53), and 1.41 (95% CI, 1.13 to 1.75), respectively, with adjustment for age, sex, birth weight, and preterm birth. In girls aged < 2 years, the overweight and obese groups also showed elevated HRs for UTIs and APN, although the underweight group had a high risk of UTIs (Table 3).

### Incidence rates of urinary tract infection, cystitis, and acute pyelonephritis according to body mass index status between 2 years and 6 years of age

**Figure 2.**
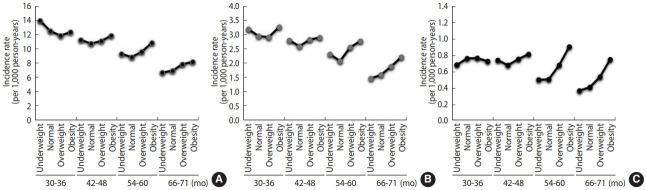
Incidence rates of (A) urinary tract infection (UTIs), (B) cystitis, and (C) acute pyelonephritis (APN), stratified by age groups and body mass index (BMI) categories. U-shaped associations between BMI categories and the incidence rates of UTIs, cystitis, and APN were found in children aged 42-48 months.

Early-life BMI between 2 years and 6 years of age showed a partial U-shaped relationship with UTI, cystitis, and APN (Figure 2). In particular, U-shaped or J-shaped associations between BMI and the IRs of UTIs, cystitis, and APN were revealed among children aged 42-48 months. The IR of UTIs was the highest in children who were underweight between 30 months and 36 months of age (n= 13.9 per 1,000 person-years), and that of UTIs decreased with age in all BMI categories. While the IR of APN decreased with age in normal-weight children, it did not decrease with age in underweight and obese children (Table 4).

## DISCUSSION

The major findings of this nationwide study are summarized as follows: (1) being underweight, overweight, or obese was associated with a higher risk of UTIs in infants and children compared to being normal-weight; (2) obese children aged 2-6 years showed elevated risks of developing APN and cystitis, while overweight children showed a higher risk of cystitis; (3) underweight boys aged 2-6 years had a high risk of UTIs and APN, while overweight and obese girls aged 2-6 years revealed elevated risks of UTI, cystitis, and APN; and (4) early-life BMI between 2 years and 6 years of age showed a partial U-shaped association with UTIs, cystitis, and APN. The IR of APN decreased with age in normal-weight children; however, it did not decrease with age in underweight and obese children between 2 years and 6 years of age.

In the present study, of 1,653,106 preschool-aged children, 2.8%, 11.5%, and 6.4% were underweight, overweight, and obese, respectively. These proportions were similar in both sexes. Although this prevalence does not reflect the entire number of existing cases of abnormal weight conditions in preschool-aged children in Korea, over 20% of enrolled children in our nationwide population-based study had an abnormal weight status. Notably, underweight, overweight, and obese children showed a higher risk of UTIs, even adjusting for confounding factors such as age, sex, birth weight, and preterm birth. The risk of UTIs was U-shaped in certain age groups, suggesting that both underweight and obesity predispose children to UTIs. In a recent meta-analysis involving 3,294 malnourished children, the pooled odds ratio for UTIs was 2.34 with respect to malnutrition, and the risk of UTIs increased with the severity of malnutrition [14]. In a systematic review of children aged 6-59 months, UTIs were found in 24.1% of severely malnourished children [21]. Moreover, in febrile children aged < 2 years, the odds ratios for UTIs in overweight and obese children relative to normal- weight children were 1.92 and 2.46, respectively [15]. In our previous single-center study involving children aged < 3 years, overweight and obese children were 1.96 times and 2.43 times more likely to have APN than controls [22]. These findings imply a U-shaped association between BMI and the UTI rate and a higher burden of UTIs in children with abnormal weight status.

The mechanisms that predispose obese individuals to infections are diverse. Obesity alters cytokine production, and the functions of natural killer and dendritic cells and macrophages are reduced during immune responses [2]. Fat accumulation disrupts lymphoid tissue integrity, and the secretion of adipocytokines is impaired in obese individuals [2]. Since an effectively functioning immune system is vital to overcome microbial assault and maintain immune homeostasis of the kidney and urinary tract, immune signaling pathway alterations could lead to the development of UTIs, causing uroepithelial tissue destruction and permanent renal damage [7,23,24]. Indeed, overweight or obesity increased the likelihood of developing UTIs, cystitis, and APN in children in the present study. Obese children aged 2-6 years showed elevated risks of APN and cystitis, and overweight children revealed a higher risk of cystitis. Overweight or obese children were found to have a higher susceptibility of developing UTIs, and more severe infections may occur in children with a higher BMI. However, this pattern was unclear in infants and children aged < 2 years. Instead, underweight children were at an elevated risk of UTIs across both age groups (< 2 and ≥ 2 years old). It is possible that underweight children are also vulnerable to infections owing to immune dysregulation. Secretory immunoglobulin A (IgA) production is impaired in malnutrition, and low secretory IgA levels in urine were found in patients with recurrent UTIs [25]. Other effects of malnutrition on the immune system comprise impaired cell-mediated immunity, phagocyte function, complement levels, and cytokine production as well as lymph node, tonsil, and thymus atrophy [26]. Reduced transferrin and increased free unbound iron levels in malnourished children may also induce a favorable environment for bacterial growth, leading to urosepsis and UTIs [26]. Our findings suggest that UTIs may develop more frequently in underweight children than in normal-weight children. Moreover, underweight and obese children did not show a decreased risk of APN with age, whereas the incidence of APN decreased with age in healthy-weight children. It is remarkable to note that underweight, as well as overweight and obese, children could be at risk of UTIs.

Furthermore, we found sex-specific differences of the incidence of UTIs, cystitis, and APN according to weight status. These findings were clearer in children aged 2-6 years, who showed a higher risk of UTIs and APN in underweight boys and a higher risk of UTIs, cystitis, and APN in overweight and obese girls. After adjusting for age, sex, birth weight, and preterm birth, underweight boys were 1.46 times more likely to have APN and obese girls were 1.41 times more likely to have APN than their normal-weight counterparts. In parallel with this finding, obese females had a 45% higher risk of UTIs, while obese males showed only a 10% higher risk in an inpatient United States database of participants aged 2-20 years [5]. As is well known, females develop UTIs more often than males since the urethral opening is closer to sources of bacteria from the vagina and anus [27]. Obesity may lead to pelvic floor, urethral, and bladder dysfunction, which places obese females at a higher risk for incontinence and thereby facilitate UTIs [28]. However, the reason for the increased risk of UTIs in underweight boys remains unclear; one possible reason may be differences in sex hormone levels and body fat distribution. Sex steroid hormones serve to regulate adipose tissue development and function [29]. The increase in body weight in boys and girls is mainly attributable to increases in lean and fat mass, respectively. Low testosterone levels were associated with lean mass loss [30]. Androgens and estrogens exert significant effects on the immune response, such as immune cell activation, proliferation, and differentiation, as well as cytokine and antibody production [31]. Thus, the decrease of lean mass in underweight boys and the increase of adipose tissue in obese girls may adversely affect the balance of sex steroid hormones, which may impair the host immune response against UTIs. Although boys and girls undergo full sexual maturation after puberty, fundamental genetic and endocrine differences exist between the sexes that shape differential immunity early in life [32]. Long before the peak in sex hormones in adolescence, a clear sexual dimorphism has been observed for susceptibility and severity in various childhood infections [33]. However, the detailed interactions of sex hormones, adipocyte function, and UTI susceptibility remain unclear.

Nutrition in early life has a major impact on long-term disease risks as well as immediate health outcomes [34]. A recent study showed that excessive weight gain at any point before 5 years of age almost entirely predicted BMI trajectories toward obesity in childhood and adolescence [35]. Dairy protein has been proposed to affect insulin-like growth factor I concentrations in infancy and to be associated with the so-called programming effects of obesity [36]. Epigenetic modifications also have been shown to be inducible by nutrient intake [34]. Many low- and middle-income countries continue to struggle against infectious diseases and undernutrition, while they are also experiencing a rapid increase in obesity and its related non-communicable diseases at the same time [37]. In the present study, being underweight, overweight, or obese under 6 years of age was associated with an increased risk for later UTIs. Although all children may not have remained in the same weight category throughout the follow-up period, abnormal weight status in the first 6 years of life may be associated with late-onset adverse health outcomes, such as UTIs, cystitis, and APN.

This study has several limitations. First, weight-for-age may not be an appropriate indicator of growth in the first 2 years of life. While it can be used to assess whether a child is underweight, it might be imprecise for classifying a child as overweight or obese. Second, there could have been some coding errors because our population-based study used administrative medical databases. Furthermore, the follow-up period was relatively short. The impact of early-life weight status on UTIs in later childhood and adolescence was not addressed in the present study. Finally, nutritional status may confound the relation between weight status and UTI risk. While BMI measurement is the most widely used method for nutritional status assessment, nutritional status may not be accurately reflected by BMI or weight status. Therefore, a cautious interpretation is needed regarding the causal relationship between weight status or BMI and UTI risk. However, the main strength of our study is that it is a large nationwide population-based study of 1,653,106 infants and children in Korea. Using the database from the NHIS and NHS for infants and children, we investigated the impact of early-life weight status on UTI development in children. Unlike other studies that have reported the UTI prevalence in the severely underweight or obese individual category, this is the first comparable analysis for the association of UTIs with a comprehensive set of BMI categories, ranging from underweight to obese, among children. The relationships between abnormal weight status and the 2 main forms of UTIs, cystitis and APN, were also analyzed. Moreover, we included data regarding potential confounding factors, such as age, sex, birth weight, and preterm birth, and these variables were adjusted using a Cox proportional hazard model.

In summary, the present study revealed that being underweight, overweight, and obese in childhood was associated with the development of UTIs, cystitis, and APN in a large Korean population. This association was evident in underweight boys and in overweight or obese girls between 2 years and 6 years of age. Body weight control will be significant for the primary prevention of UTIs, cystitis, and APN in a given population. Further analyses should be performed to determine whether an increase or a reduction in body weight will prevent the occurrence of UTIs in children with abnormal early-life weight status.

## Figures and Tables

**Table 1.** Baseline characteristics and incidence of outcomes according to weight status categories^1^

Characteristics	Underweight	Normal	Overweight	Obesity	p-value^2^
Total	46,744 (2.8)	1,310,102 (79.3)	190,735 (11.5)	105,525 (6.4)	-
Age (mo)	36.3±20.1	29.0±21.5	27.3±22.4	25.6±23.2	<0.001
Sex (male)	22,305 (47.7)	653,518 (49.9)	100,594 (52.7)	55,317 (52.4)	<0.001
Preterm birth	2,960 (6.3)	35,229 (2.7)	3,559 (1.9)	1,901 (1.8)	<0.001
Birthweight (kg)	2.92±0.49	3.17±0.44	3.33±0.43	3.40±0.44	<0.001
UTI	2,384 (5.1)	73,792 (5.6)	11,357 (5.9)	6,652 (6.3)	<0.001
APN	190 (0.4)	7,075 (0.5)	1,217 (0.6)	759 (0.7)	<0.001
Cystitis	467 (1.0)	13,077 (1.0)	2,018 (1.1)	1,154 (1.1)	<0.001

Values are presented as number (%) or mean±standard deviation. UTI, urinary tract infection; APN, acute pyelonephritis.

1 Weight-for-age category for infants aged 4-24 months and body mass index category for children aged 2-6 years; Weight status was significantly associated with the total number of cases of UTIs, APN, and cystitis without adjustment for confounding variables.

2 Analyses of variance was used for continuous variables and the chi-square test for categorical variables.

**Table 2.** Hazard ratios^1^ for the development of UTIs according to body weight status^2^

Variables		n	UTI	Incidence duration^3^	IR^4^	Model 1	Model 2	Model 3	Cystitis	Incidence duration^3^	IR^4^	Model 1	Model 2	Model 3	APN	Incidence duration^3^	IR^4^	Model 1	Model 2	Model 3
Total																				
	Under	46,744	2,384	148,648	16.04	0.90 (0.86, 0.93)	1.07 (1.03, 1.12)	1.05 (1.01, 1.10)	467	152,484	3.06	0.99 (0.91, 1.09)	1.07 (0.98, 1.18)	1.06 (0.97, 1.16)	190	152,940	1.24	0.75 (0.65, 0.87)	0.99 (0.85, 1.15)	0.98 (0.85, 1.14)
	Normal	1,310,102	73,792	4,108,381	17.96	1.00 (reference)	1.00 (reference)	1.00 (reference)	13,077	4,235,069	3.09	1.00 (reference)	1.00 (reference)	1.00 (reference)	7,075	4,243,133	1.67	1.00 (reference)	1.00 (reference)	1.00 (reference)
	Over	190,735	11,357	597,163	19.02	1.06 (1.04, 1.08)	1.02 (1.0, 1.04)	1.02 (1.00, 1.04)	2,018	616,804	3.27	1.06 (1.01, 1.11)	1.05 (1.00, 1.10)	1.06 (1.01, 1.11)	1,217	617,875	1.97	1.18 (1.11, 1.26)	1.11 (1.05, 1.18)	1.11 (1.04, 1.18)
	Ob	105,525	6,652	327,110	20.34	1.13 (1.10, 1.16)	1.03 (1.01, 1.06)	1.04 (1.02, 1.07)	1,154	338,768	3.41	1.10 (1.04, 1.17)	1.07 (1.01, 1.14)	1.08 (1.01, 1.14)	759	339,160	2.24	1.34 (1.24, 1.44)	1.17 (1.08, 1.26)	1.16 (1.08, 1.25)
4 mo-2 yr																				
	Under	12,397	1,119	36,271	30.85	1.09 (1.03, 1.16)	1.13 (1.07, 1.20)	1.10 (1.04, 1.17)	164	38,315	4.28	1.10 (0.94, 1.28)	1.10 (0.94, 1.28)	1.07 (0.92, 1.25)	120	38,337	3.13	1.03 (0.86, 1.23)	1.09 (0.91, 1.30)	1.07 (0.89, 1.28)
	Normal	587,583	48,774	1,730,102	28.19	1.00 (reference)	1.00 (reference)	1.00 (reference)	7,088	1,820,608	3.89	1.00 (reference)	1.00 (reference)	1.00 (reference)	5,537	1,820,395	3.04	1.00 (reference)	1.00 (reference)	1.00 (reference)
	Over	98,265	8,147	290,252	28.07	1.00 (0.98, 1.02)	0.99 (0.96, 1.01)	0.99 (0.97, 1.02)	1,200	305,486	3.93	1.01 (0.95, 1.07)	1.02 (0.96, 1.09)	1.03 (0.96, 1.09)	998	305,362	3.27	1.08 (1.01, 1.15)	1.06 (0.99, 1.14)	1.06 (0.99, 1.14)
	Ob	60,261	5,005	176,913	28.29	1.00 (0.97, 1.03)	0.97 (0.95, 1.00)	0.98 (0.95, 1.01)	727	186,296	3.90	1.00 (0.93, 1.08)	1.01 (0.93, 1.08)	1.01 (0.93, 1.09)	637	186,133	3.42	1.12 (1.04, 1.22)	1.08 (0.99, 1.17)	1.08 (0.99, 1.17)
2-6 yr																				
	Under	34,347	1,265	112,377	11.26	1.07 (1.01, 1.13)	1.06 (1.01, 1.13)	1.05 (1.00, 1.11)	303	114,169	2.65	1.07 (0.95, 1.20)	1.06 (0.94, 1.19)	1.05 (0.94, 1.18)	70	114,603	0.61	0.96 (0.75, 1.22)	0.95 (0.75, 1.21)	0.96 (0.76, 1.22)
	Normal	722,519	25,018	2,378,280	10.52	1.00 (reference)	1.00 (reference)	1.00 (reference)	5,989	2,414,462	2.48	1.00 (reference)	1.00 (reference)	1.00 (reference)	1,538	2,422,738	0.63	1.00 (reference)	1.00 (reference)	1.00 (reference)
	Over	92,470	3,210	306,912	10.46	1.00 (0.96, 1.03)	1.03 (0.99, 1.07)	1.03 (1.00, 1.07)	818	311,317	2.63	1.06 (0.99, 1.14)	1.09 (1.02, 1.18)	1.10 (1.02, 1.18)	219	312,513	0.70	1.11 (0.96, 1.28)	1.14 (0.99, 1.31)	1.12 (0.97, 1.30)
	Ob	45,264	1,647	150,197	10.97	1.05 (1.00, 1.10)	1.11 (1.05, 1.16)	1.11 (1.06, 1.17)	427	152,472	2.80	1.13 (1.03, 1.25)	1.19 (1.08, 1.32)	1.20 (1.09, 1.32)	122	153,027	0.80	1.26 (1.05, 1.52)	1.32 (1.10, 1.59)	1.30 (1.08, 1.57)

Values are presented as hazard ratio (95% confidence interval). Model 1, unadjusted; model 2, adjusted for age and sex; model 3, adjusted for age, sex, birth weight, and preterm birth. UTI, urinary tract infection; IR, incidence rate; APN, acute pyelonephritis; Under, underweight; Over, overweight; Ob, obese.

1 Cox proportional hazard model analysis was conducted for the outcome (UTI, cystitis, and APN).

2 Weight-for-age categories for infants aged 4-24 months and body mass index categories for children aged 2-6 years.

3 Units: person-years.

4 The IR is per 1,000 person-years.

**Table 3.** Hazard ratio^1^ for the development of UTIs according to body weight status^2^ and sex

Variables			n	UTI	Incidence duration^3^	IR^4^	Model 1	Model 2	Model 3	Cystitis	Incidence duration^3^	IR^4^	Model 1	Model 2	Model 3	APN	Incidence duration^3^	IR^4^	Model 1	Model 2	Model 3
4 mo-2 yr																					
	Male																				
		Under	5,419	464	15,899	29.18	1.06 (0.94, 1.16)	1.11 (1.01, 1.21)	1.07 (0.97, 1.17)	65	16,773	3.88	1.14 (0.89, 1.45)	1.16 (0.91, 1.48)	1.10 (0.86, 1.41)	52	16,750	3.10	1.11 (0.84, 1.46)	1.21 (0.92, 1.59)	1.18 (0.89, 1.55)
		Normal	289,974	23,560	853,824	27.59	1.00 (reference)	1.00 (reference)	1.00 (reference)	3,068	899,090	3.41	1.00 (reference)	1.00 (reference)	1.00 (reference)	2,512	898,561	2.80	1.00 (reference)	1.00 (reference)	1.00 (reference)
		Over	55,145	4,351	162,989	26.70	0.97 (0.94, 1.00)	0.95 (0.92, 0.98)	0.96 (0.92, 0.99)	560	171,399	3.27	0.96 (0.88, 1.05)	0.95 (0.87, 1.04)	0.96 (0.88, 1.05)	473	171,308	2.76	0.99 (0.90, 1.09)	0.95 (0.86, 1.05)	0.95 (0.86, 1.05)
		Ob	33,223	2,614	97,793	26.73	0.97 (0.93, 1.01)	0.94 (0.90, 0.98)	0.95 (0.91, 0.99)	335	102,888	3.26	0.95 (0.85, 1.07)	0.94 (0.84, 1.05)	0.96 (0.85, 1.07)	280	102,795	2.72	0.97 (0.86, 1.10)	0.92 (0.81, 1.04)	0.93 (0.82, 1.05)
	Female																				
		Under	6,978	655	20,372	32.15	1.11 (1.03, 1.20)	1.15 (1.07, 1.24)	1.13 (1.04, 1.22)	99	21,542	4.60	1.05 (0.86, 1.28)	1.07 (0.87, 1.3)	1.06 (0.87, 1.30)	68	21,587	3.15	0.96 (0.75, 1.22)	1.01 (0.80, 1.29)	1.00 (0.79, 1.28)
		Normal	297,609	25,214	876,278	28.77	1.00 (reference)	1.00 (reference)	1.00 (reference)	4,020	921,518	4.36	1.00 (reference)	1.00 (reference)	1.00 (reference)	3,025	921,835	3.28	1.00 (reference)	1.00 (reference)	1.00 (reference)
		Over	43,120	3,796	127,263	29.83	1.04 (1.00, 1.08)	1.03 (1.00, 1.07)	1.04 (1.00, 1.07)	640	134,087	4.77	1.10 (1.01, 1.19)	1.09 (1.00, 1.19)	1.09 (1.00, 1.18)	525	134,053	3.92	1.20 (1.09, 1.31)	1.18 (1.08, 1.30)	1.18 (1.07, 1.29)
		Ob	27,038	2,391	79,120	30.22	1.05 (1.01, 1.09)	1.02 (0.97, 1.06)	1.02 (0.98, 1.07)	392	83,408	4.70	1.08 (0.97, 1.20)	1.07 (0.96, 1.18)	1.06 (0.95, 1.17)	357	83,338	4.28	1.30 (1.17, 1.45)	1.24 (1.11, 1.38)	1.23 (1.10, 1.37)
2-6 yr																					
	Male																				
		Under	16,886	570	55,361	10.30	1.10 (1.01, 1.20)	1.11 (1.02, 1.21)	1.10 (1.01, 1.19)	117	56,179	2.08	1.12 (0.93, 1.35)	1.13 (0.94, 1.36)	1.12 (0.93, 1.35)	34	56,327	0.60	1.42 (1.00, 2.00)	1.42 (1.01, 2.01)	1.46 (1.03, 2.07)
		Normal	363,544	11,157	1,198,212	9.31	1.00 (reference)	1.00 (reference)	1.00 (reference)	2,255	1,215,146	1.86	1.00 (reference)	1.00 (reference)	1.00 (reference)	517	1,218,312	0.42	1.00 (reference)	1.00 (reference)	1.00 (reference)
		Over	45,449	1,241	151,327	8.20	0.88 (0.83, 0.94)	0.92 (0.87, 0.97)	0.92 (0.87, 0.98)	260	153,165	1.70	0.92 (0.81, 1.04)	0.95 (0.83, 1.08)	0.95 (0.84, 1.08)	58	153,574	0.38	0.89 (0.68, 1.17)	0.92 (0.70, 1.20)	0.90 (0.68, 1.18)
		Ob	22,094	612	73,627	8.31	0.90 (0.83, 0.97)	0.96 (0.88, 1.04)	0.96 (0.89, 1.05)	118	74,537	1.58	0.86 (0.71, 1.03)	0.90 (0.75, 1.09)	0.91 (0.75, 1.09)	34	74,688	0.46	1.08 (0.76, 1.52)	1.13 (0.80, 1.60)	1.10 (0.78, 1.56)
	Female																				
		Under	17,461	695	57,016	12.19	1.04 (0.96, 1.12)	1.03 (0.95, 1.11)	1.02 (0.95, 1.10)	186	57,990	3.21	1.03 (0.89, 1.19)	1.02 (0.88, 1.18)	1.02 (0.88, 1.18)	36	58,276	0.62	0.73 (0.52, 1.01)	0.72 (0.52, 1.01)	0.73 (0.52, 1.02)
		Normal	358,975	13,861	1,180,068	11.75	1.00 (reference)	1.00 (reference)	1.00 (reference)	3,734	1,199,315	3.11	1.00 (reference)	1.00 (reference)	1.00 (reference)	1,021	1,204,426	0.85	1.00 (reference)	1.00 (reference)	1.00 (reference)
		Over	47,021	1,969	155,585	12.66	1.08 (1.03, 1.13)	1.11 (1.06, 1.17)	1.12 (1.07, 1.17)	558	158,152	3.53	1.14 (1.04, 1.24)	1.18 (1.08, 1.29)	1.18 (1.08, 1.29)	161	158,940	1.01	1.20 (1.02, 1.42)	1.24 (1.05, 1.47)	1.23 (1.04, 1.46)
		Ob	23,170	1,035	76,571	13.52	1.15 (1.08, 1.23)	1.22 (1.14, 1.30)	1.22 (1.15, 1.31)	309	77,935	3.96	1.28 (1.14, 1.43)	1.36 (1.21, 1.53)	1.37 (1.21, 1.53)	88	78,339	1.12	1.33 (1.07, 1.65)	1.42 (1.14, 1.77)	1.41 (1.13, 1.75)

Values are presented as hazard ratio (95% confidence interval). Model 1, unadjusted; model 2, adjusted for age and sex; model 3, adjusted for age, sex, birth weight, and preterm birth. UTI, urinary tract infection; IR, incidence rate; APN, acute pyelonephritis; Under, underweight; Over, overweight; Ob, obese.

1 Cox proportional hazard model analysis was conducted for the outcome (UTI, cystitis, and APN).

2 Weight-for-age categories for infants aged 4-24 months and body mass index categories for children aged 2-6 years.

3 Units: person-years.

4 The IR is per 1,000 person-years.

**Table 4.** IRs of UTIs according to the examination periods of NHS for infants and children

Variables	BMI	n	UTI	Incidence duration^1^	IR^2^	Cystitis	Incidence duration^1^	IR^2^	APN	Incidence duration^1^	IR^2^
30-36 mo (4th)	Underweight	14,053	624	44,776	13.94	145	45,654	3.18	31	45,838	0.68
	Normal	289,179	11,532	924,025	12.48	2,765	940,654	2.94	714	944,317	0.76
	Overweight	26,832	1,022	86,204	11.86	254	87,610	2.90	67	87,964	0.76
	Obesity	11,397	452	36,652	12.33	121	37,294	3.24	27	37,466	0.72
42-48 mo (5th)	Underweight	8,825	330	29,401	11.22	83	29,859	2.78	22	29,987	0.73
	Normal	196,682	7,096	660,065	10.75	1,728	670,450	2.58	453	672,859	0.67
	Overweight	30,070	1,124	101,180	11.11	288	102,773	2.80	77	103,215	0.75
	Obesity	13,293	529	44,732	11.83	131	45,505	2.88	37	45,676	0.81
54-60 mo (6th)	Underweight	6,540	203	21,877	9.28	51	22,178	2.30	11	22,261	0.49
	Normal	138,483	4,133	467,418	8.84	978	473,373	2.07	237	474,874	0.50
	Overweight	21,587	701	73,079	9.59	188	74,016	2.54	50	74,315	0.67
	Obesity	11,338	415	38,213	10.86	107	38,791	2.76	35	38,913	0.90
66-71 mo (7th)	Underweight	4,933	109	16,333	6.67	24	16,491	1.46	6	16,529	0.36
	Normal	98,302	2,263	327,155	6.91	520	330,376	1.57	134	331,084	0.40
	Overweight	14,005	365	46,515	7.85	88	46,990	1.87	25	47,090	0.53
	Obesity	9,246	251	30,633	8.19	68	30,915	2.20	23	31,004	0.74

IR, incidence rate; UTI, urinary tract infection; NHS, National Health Screening program; BMI, body mass index; APN, acute pyelonephritis.

1 Units: person-years.

2 The IR is per 1,000 person-years.
